# Two pathologically confirmed cases of novel mutations in the *MAPT* gene causing frontotemporal dementia

**DOI:** 10.1016/j.neurobiolaging.2019.11.009

**Published:** 2020-03

**Authors:** Rachelle Shafei, Ione O.C. Woollacott, Catherine J. Mummery, Martina Bocchetta, Rita Guerreiro, Jose Bras, Jason D. Warren, Tammaryn Lashley, Zane Jaunmuktane, Jonathan D. Rohrer

**Affiliations:** a Dementia Research Centre, Department of Neurodegenerative Disease, UCL Queen Square Institute of Neurology, University College London, London, UK; b Center for Neurodegenerative Science, Van Andel Institute, Grand Rapids, MI, USA; c Division of Psychiatry and Behavioral Medicine, Michigan State University College of Human Medicine, Grand Rapids, MI, USA; d Queen Square Brain Bank for Neurological Disorders, Department of Clinical and Movement Neurosciences, UCL Queen Square Institute of Neurology, London, UK; e Department of Neurodegenerative Disease, UCL Queen Square Institute of Neurology, University College London, London, UK

**Keywords:** Tau, Frontotemporal dementia, MAPT

## Abstract

*MAPT* mutations were the first discovered genetic cause of frontotemporal dementia (FTD) in 1998. Since that time, over 60 *MAPT* mutations have been identified, usually causing behavioral variant FTD and/or parkinsonism clinically. We describe 2 novel *MAPT* mutations, D252V and G389_I392del, each presenting in a patient with behavioral variant FTD and associated language and cognitive deficits. Neuroimaging revealed asymmetrical left greater than right temporal lobe atrophy in the first case, and bifrontal atrophy in the second case. Disease duration was 8 years and 5 years, respectively. Postmortem examination in both patients revealed a 3-repeat predominant tauopathy, similar in appearance to Pick's disease. These 2 mutations add to the literature on genetic FTD, both presenting with similar clinical and imaging features to previously described cases, and pathologically showing a primary tauopathy similar to a number of other *MAPT* mutations.

## Highlights

• Over 60 *MAPT* mutations are currently described—we describe 2 novel mutations: p.D252V and p.G389_I392del.
• Both novel mutations are associated with a frontotemporal dementia clinical syndrome similar to previously described *MAPT* mutations.
• Both the novel mutations are associated with a primary 3-repeat tauopathy at postmortem.

## 1 Background

Frontotemporal dementia (FTD) is a clinically and pathologically heterogeneous group of neurodegenerative disorders. Around one-third of FTD is familial, with the first gene identified as having causative mutations being microtubule-associated protein tau (*MAPT*) in 1998 (Hutton et al., 1998, Poorkaj et al., 1998, Spillantini et al., 1998). Since that time, over 60 *MAPT* mutations have been discovered, with the majority presenting with clinical features of behavioral change and/or parkinsonism (Greaves and Rohrer, 2019). Other symptoms include semantic impairment (although it is rare for language problems to be the presenting feature) and, unlike most other forms of FTD, episodic memory difficulties (Liang et al., 2014, Tolboom et al., 2010). Age of symptom onset is variable (although is commonly in the 40s or 50s), as is disease duration, which can vary between a few years and over twenty (Ygland et al., 2018). Pathologically, there is also heterogeneity: often both 3-repeat (3R) and 4-repeat (4R) isoforms of tau are found in the neuronal and glial inclusions at postmortem but for some mutations 3R-tau, inclusions are predominant while others have predominantly 4R-tau (Ghetti et al., 2015). In this case series, we report 2 individuals who presented with FTD and were found to have novel pathogenic *MAPT* mutations with subsequent pathological confirmation of a primary tauopathy.

## 2 Methods

### 2.1 Clinical

Both patients attended the Specialist Cognitive Disorders Clinic at the National Hospital for Neurology and Neurosurgery where they underwent a standard clinical and neuropsychological assessment. Magnetic resonance imaging was performed on a 3T Siemens Trio scanner.

### 2.2 Genetics

Blood was taken for genetic testing in both patients with informed consent. Sequencing of exons 1 and 9–13 of the *MAPT* gene was performed initially. Subsequently both samples underwent exome sequencing. Exome enrichment was performed using TruSeq Exome Capture kit (Illumina, San Diego, CA, USA). Sequencing was performed on a HiSeq 2000 (Illumina). Reads were aligned to GRCh37/hg19 using BWA, variants called according to GATK best practice guidelines and annotated with ANNOVAR (Wang et al., 2010). In silico pathogenicity predictions of nonsynonymous variants were done with the combined annotation-dependent depletion (CADD) (Kircher et al., 2014). Variants were filtered against population databases (1000Genomes, ESP, and gnomAD) and assessed based on variant type (missense, nonsense, splice site, frameshift, nonframeshift) and predicted pathogenicity.

### 2.3 Neuropathology

Both patients consented to brain donation at the Queen Square Brain Bank for Neurological Disorders (QSBB). After death, the brains were processed according to QSBB protocol, which involves freezing of the right brain hemisphere, and fixation in 10% buffered formalin with tissue sampling and processing for paraffin histology of the left hemisphere. All tissues are stored at QSBB under a licence from the Human Tissue Authority. Sections of 7 μm thickness were cut from formalin-fixed paraffin-embedded tissue blocks from multiple brain regions, mounted on glass slides and stained with hematoxylin and eosin. Representative sections were examined with immunohistochemical stains with the following antibodies: phospho-tau (AT8 MN1020, 1:600; Thermo), Aβ (6F3D, 1:100; Dako), α-synuclein (ab80627, 1:1500; Abcam), TDP-43 (2E2-D3, 1:5000; Abnova), 4R tau (1E1/A6, 1:4000; Millipore), and 3R tau (8E6/C11, 1:800; Millipore). All immunostainings were carried out on an automated immunostaining machine (A.Menarini Diagnostics) following manufacturer's guidelines using 3,3′-diaminobenzidine as chromogen and appropriate positive and negative controls. Ethical approval for the study was obtained from the Local Research Ethics Committee of the National Hospital for Neurology and Neurosurgery.

## 3 Results

### 3.1 Case 1

#### 3.1.1 Clinical history

A 46-year-old right-handed gentleman presented to his local neurologist with a one-year history of behavioral change and impairment of language and cognition. He had become more socially withdrawn and apathetic with poor self-care. He had also become more obsessive with a preference for routines. Over the same period, he had had difficulty with understanding the meaning of words and had developed difficulties with recognizing both faces and familiar places, the latter impairment leading to him getting lost at times. He was otherwise fit and well without any other medical problems.

The presentation led to a referral to our clinic where he underwent a detailed cognitive assessment. His speech was noted to be fluent but empty of content with repeated use of stock phrases. MMSE score was 22/30. He was anomic with poor single word comprehension and a surface dyslexia.

Neuropsychometric testing revealed a verbal IQ of 73 and performance IQ of 125. He scored below the 5th percentile on the Oldfield Naming Test. He named only 2 animal and 7 F words on testing verbal fluency. Posterior cortical function was intact, scoring full marks on the Visual Object Space and Perception battery Incomplete Letters and Cube Analysis subtests. Executive function testing revealed performance within the normal range on the Brixton Spatial Anticipation task (75–90th percentile) and the Trail Making Test Part B (25–50th percentile).

There was a strong family history of dementia with his mother developing “dementia” in her 70s, and all but one of her 7 siblings also developing “dementia” in their 60s or 70s. Little was known about the phenotype of his relatives.

**Figure 1:**
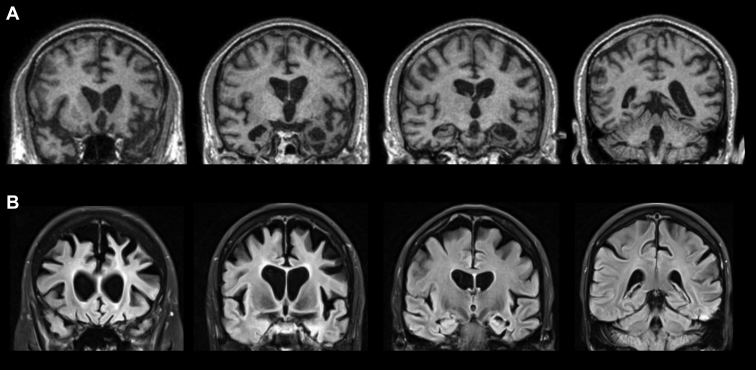
Magnetic resonance imaging of case 1 (A) and case 2 (B) showing coronal volumetric T1 (case 1) and FLAIR (case 2) scans. Asymmetrical, left more than right, temporal greater than frontal atrophy is seen in case 1, whereas in case 2, there is relatively symmetrical bilateral frontal more than temporal lobe atrophy.

MR imaging (Fig 1A) showed bilateral anterior temporal lobe atrophy, particularly affecting the medial temporal lobes including the amygdala and anterior hippocampus, more marked on the left than the right. There was less marked frontal lobe involvement, but particularly involving the left orbitofrontal cortex.

A diagnosis of FTD with both behavioral symptoms and prominent semantic impairment was made at this time.

He was reviewed twice over the next year. Worsening behavior was noted with hoarding, development of a sweet tooth and inappropriate trusting behavior. Comprehension had deteriorated with difficulties communicating effectively by the last appointment. He was not seen again in the clinic, requiring full-time care from this point. He died 5 years later at the age of 54 years.

#### 3.1.2 Genetics

The patient was found to carry a novel missense variant in exon 9 of the *MAPT* gene: NM_001123066.3: c.1760A > T: p.Asp587Val. In some nomenclatures of the *MAPT* gene, this is known as p.D252V. He subsequently underwent exome sequencing and no other mutations were found in any of the genes known to cause frontotemporal dementia apart from a variant of uncertain significance in *DCTN1* (NM_004082.4: c.2264dupG: p.Ser755fs). Using CADD to predict the deleteriousness of the *MAPT* variant, we obtained a normalized PHRED score of 21.5, indicating this variant is predicted to be in the top 1% of the most deleterious variants in the human genome.

#### 3.1.3 Neuropathology

**Fig. 2:**
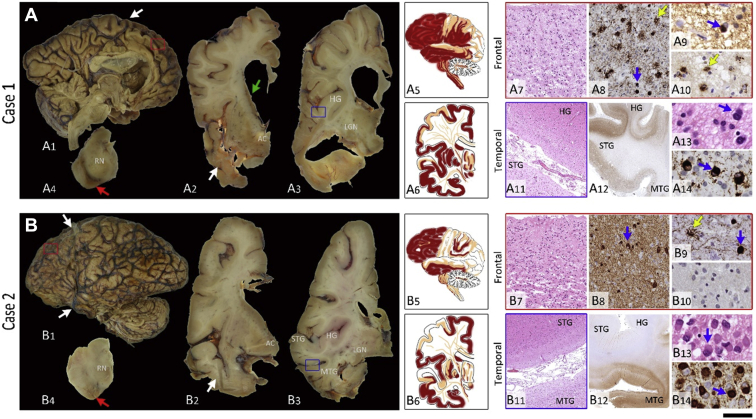
Macroscopic and microscopic pathology and schematic representation of case 1 (A1–A14) and case 2 (B1–B14). Prominent frontal lobe atrophy is seen on the medial surface in case 1 (A1) and on the convex lateral surface in case 2 (B1) (sharp, knife-edge frontal lobe atrophy with some sparing of the motor cortex in both cases is highlighted with white arrows). Frontal lobe atrophy is also evident on the coronal sections of both cases (A2, A3, B2, and B3). The anterior temporal lobe at the level of the anterior commissure (AC) shows particularly severe atrophy in case 1 (A2, white arrow), and comparably less severe medial atrophy with better preservation of the superior anterior temporal gyrus in case 2 (B2, white arrow). At the level of the lateral geniculate nucleus (LGN) in case 1, there is very severe atrophy of the hippocampus, parahippocampal and inferior temporal gyri, middle temporal gyrus (MTG), and superior temporal gyrus (STG), with better preservation of the Heschl's gyrus (HG) (A3). In case 2, there is prominent atrophy of the hippocampus, parahippocampal, inferior and middle temporal gyri, with much better preservation of the STG and HG (B3). The frontal horns of the lateral ventricles are severely dilated in both cases (A1, A2, A3, B2, and B3) and the temporal horn is severely dilated in case 1 (A3). Also, the 3rd ventricles are dilated in both cases (A3 and B3). In case 1, there is marked atrophy of the caudate nucleus (A2, green arrow) and in both cases, there is globus pallidus (A2 and B2) and mild thalamus atrophy (A3 and B3). In the midbrain at the level of the red nucleus (RN) in both cases, there is conspicuous atrophy of the frontopontine tracts (A4 and B4, red arrows,) and pallor of the substantia nigra, which shows rusty discoloration in case 1. Schematic representation of the extent of atrophy and tau pathology severity is demonstrated for case 1 (A5 and A6) and case 2 (B5 and B6, dark red represents the most severe pathology, amber scanty atrophy and tau pathology, and uncolored corresponds to areas with preserved cytoarchitecture and absent tau pathology). Very severe pan-cortical frontal lobe atrophy with rarefaction of the neuropil and prominent gliosis is confirmed in both cases (A7 and B7, corresponding regions highlighted as red squares in A1 and B1). In case 1, across the frontal lobe, in addition to Pick bodies (A8, blue arrow), there is widespread cortical astrocytic tau pathology, resembling ramified and tufted astrocytes (yellow arrow). Pick bodies show positive immunolabeling for 3R tau (A9, blue arrow) and a proportion of the astrocytic tau pathology is immunoreactive for 4R tau (A10, yellow arrow). In the frontal lobe of case 2, there is a dense meshwork of tau positive neuropil threads and frequent Pick bodies (B8, blue arrow). 3R tau immunostaining accentuates Pick bodies (B9, blue arrow) and also highlights occasional astrocytic tau pathology (yellow arrow), while immunostaining for 4R tau (B10) is negative. In case 1, there is severe atrophy of STG and better preserved cortical cytoarchitecture in HG (A11). In case 2, MTG shows severe atrophy, but the cytoarchitecture of STG is much better preserved (B11, corresponding macroscopic regions are highlighted with blue squares in A3 and B3). Density of hyper-phosphorylated tau pathology in the medial temporal lobes of both cases reflects the degree of cortical atrophy; in case 1, tau pathology extends into HG (A12), but in case 2, tau pathology is minimal in the superior aspect of the STG and in HG (B12). In case 1, there is a very severe atrophy of the dentate gyrus with tau-positive Pick bodies seen in the residual granule cells (A13 and A14, blue arrows). Granule cells of the dentate gyrus in case 2 are comparably better preserved, with numerous Pick bodies evident in the residual neurones (B13 and B14, blue arrows highlight representative inclusions). Scale bar: 300 μm in A11 and B11; 600 μm A12 and B12; 100 μm in A7, A8, B7, and B8; 50 μm in A9, A10, B9, and B10; and 30 μm in A13, A14, B13, and B14. (For interpretation of the references to color in this figure legend, the reader is referred to the Web version of this article.)

Macroscopically, with a whole brain weight of approximately 830 g, there was global atrophy with emphasis in the temporal and frontal lobes, with widespread cortical thinning, reduction in white matter bulk, and severe dilation of the lateral and 3rd ventricles (Fig. 2, A1–A3). The caudate nucleus showed severe atrophy and the globus pallidus was darkly discolored while the putamen was only mildly reduced in size (Fig. 2, A2). The thalamus showed a reduction in size (Fig. 2, A3), but the subthalamic nucleus appeared normal. There was very severe atrophy of the hippocampus and the amygdala was not discernible because of the extremely severe atrophy (Fig. 2, A3). The size of the brain stem was reduced, and in the midbrain, there was particularly prominent atrophy of the frontopontine tracts and pallor of the substantia nigra (Fig. 2, A4). The locus coeruleus was well pigmented. The cerebellum showed no macroscopic abnormality.

Microscopically, there was severe pan-cortical atrophy, with spongiosis, marked chronic gliosis, and severe neuronal loss in the frontal and temporal lobes, including superior temporal gyrus (Fig. 2 A7 and A11). In the parietal lobe (inferior parietal lobule more than superior parietal lobule), there was prominent pan-cortical atrophy. Tau immunohistochemistry revealed a meshwork of neuropil threads, pretangles, globular inclusions (Pick bodies), and neurofibrillary tangles as well as widespread pan-cortical astrocytic tau pathology in the gray matter of frontal, temporal, and parietal lobes (Fig. 2 A8 and A12) with a fine meshwork of threads and occasional coiled bodies in the white matter. Most neuronal cytoplasmic and thread tau pathology was confirmed to be 3R-tau, whereas a proportion of the astrocytic tau pathology showed labeling with antibodies against 4R-tau (Fig. 2, A9–A10). Microscopic examination confirmed the macroscopically evident very severe atrophy of the hippocampal subregions and the amygdala atrophy with particularly widespread atrophy of the dentate gyrus. Across the medial temporal lobe, there were phospho-tau (AT8) and 3R-tau positive neuropil threads, Pick bodies, neurofibrillary tangles, and pretangles (Fig. 2, A13–A14). In the severely atrophic caudate, the less atrophic globus pallidus, putamen, and thalamus, there was a dense phospho-tau and 3R-tau immunoreactive neuropil thread and globular and globose neuronal cytoplasmic tau pathology. Severe neuronal loss with free pigment deposition and prominent chronic gliosis was seen in the substantia nigra accompanied by dense phospho-tau immunoreactive thread and tangle pathology across the midbrain. The corticospinal tracts across the brain stem showed severe atrophy. While the overall cerebellar cytoarchitecture was preserved, scanty tau pathology was seen in the cerebellar white matter, with more frequent threads and pretangles evident across the dentate nucleus. Histological examination revealed no evidence of Aβ, α-synuclein, or TDP-43 pathology and assessment of Braak & Braak neurofibrillary tangle stage was not feasible because of the presence of the primary tauopathy.

### 3.2 Case 2

#### 3.2.1 Clinical history

A 49-year-old woman presented to our clinic with a 2-year history of behavioral change, language impairment, and poor planning and decision-making. She had become more apathetic with a decline in self-care. There was increasing word-finding difficulty with problems at home suggestive of executive dysfunction, for example, difficulties following instructions and difficulty operating the cooker.

The patient's mother died in middle age without a diagnosis of dementia, although aggressive behavior was reported. However, her mother's mother developed dementia with behavioral change in her early 50s, dying at the age of 70 years.

On examination, speech was fluent but with clear word-finding difficulties. MMSE score was 16/30. Executive function was impaired with poor cognitive estimates and concrete interpretation of proverbs.

Neuropsychometric testing revealed a verbal IQ of 54 and a performance IQ of 60. She named only 3 animal and 2 S words on testing verbal fluency. She was globally impaired with problems in episodic memory (<5th percentile on the Topographical Recognition Memory Test), naming (<5th percentile on the Oldfield Naming Test), visuospatial skills (<5th percentile on the Position Discrimination task of the Visual Object Space and Perception battery), and executive function (failure on the Weigl Sorting Task). Visuoperceptual skills however were relatively intact (normal performance on the Incomplete Letters task of the Visual Object Space and Perception battery).

MR imaging (Fig. 1B) showed bilateral, relatively symmetrical, frontal (mainly dorsolateral prefrontal cortex) more than temporal lobe atrophy.

A diagnosis of FTD with both behavioral symptoms and severe cognitive difficulties was made at this time.

She was reviewed one year later when her speech had deteriorated with severe word-finding difficulties and minimal speech. Behavior had deteriorated with increasing emotional lability.

She was then seen a further 2 years later by which time she required full-time care and was doubly incontinent with impaired mobility. She was not seen again in the clinic and died 2 years later at the age of 54 years.

#### 3.2.2 Genetics

The patient was found to carry a novel variant in exon 13 of the *MAPT* gene: NM_001123066.3: c.2171_2182del: p.Gly724_Ile727del. In some nomenclatures of the *MAPT* gene, this is known as p.G389_I392del. This is a heterozygous deletion of 12 nucleotides leading to a deletion of 4 amino acids. Conservation assessment of the deleted region using phastCons produced scores between 0.901 and 1 reflecting a high probability of the deleted nucleotides belonging to a conserved element of the genome. Using CADD to predict the deleteriousness of this variant, we obtained a normalized Phred score of 21.5, indicating this variant is predicted to be in the top 1% of the most deleterious variants in the human genome. Of note, 2 point mutations, both reported to be pathogenic, have been previously described in a residue included in this deletion: NM_001123066.3:c.2170G > A:p.Gly724Arg (or p.G387R: Pickering-Brown et al., 2000, Bermingham et al., 2008), and NM_001123066.3:c.2170G > C: p.Gly724Arg (or p.G387R: Murrell et al., 1999, Ghetti et al., 2000).

#### 3.2.3 Neuropathology

Macroscopically, with a whole brain weight of approximately 970g, there was severe frontal and anterior and medial temporal lobe atrophy with a thin and discolored cortical ribbon (Fig. 2, B1–B3). There was also prominent atrophy of the inferior parietal lobule and enlargement of the frontal horn of the lateral ventricle, and to a lesser extent, the 3rd ventricle. All deep brain nuclei were mildly reduced in size (Fig. 2, B2, B3). Prominent atrophy of the amygdala and hippocampus was observed, but the superior temporal and Heschl's gyri were preserved in bulk (Fig. 2, B3). The bulk of the brain stem was reduced and the frontopontine tracts in the midbrain and corticospinal tracts in the pons showed marked atrophy. The substantia nigra showed pallor rostrally at the level of the red nucleus (Fig. 2, B4), and also locus coeruleus was pale. The cerebellum was unremarkable.

Microscopically, there was very severe pan-cortical atrophy of the anterior frontal lobe, middle temporal (but not superior temporal or Heschl's) gyri (Fig. 2, B7 and B11), and inferior parietal lobule. Immunostaining for phospho-tau (AT8) confirmed a primary tauopathy with a dense meshwork of pan-cortical neuropil threads and frequent globular 3R-tau immunoreactive neuronal inclusions (Pick bodies) across the frontal, temporal, and parietal lobes. 3R-tau immunostaining also highlighted occasional astrocytic inclusions, whereas 4R-tau-positive inclusions were absent (Fig. 2, B8, B9, B10, and B12). Tau pathology in the form of scattered threads and occasional coiled bodies was also seen in the subcortical white matter of frontal, temporal, and parietal lobes. The atrophic hippocampus and amygdala contained dense tau-positive thread and tangle pathology with frequent 3R-tau-positive threads and Pick bodies and globose tangles in the residual neurons (Fig. 2, B13 and B14). Dense neuropil thread and frequent neuronal tangle and Pick body pathology was seen throughout the caudate, Meynert nucleus, internal part of the globus pallidus, and medial aspect of the putamen, with comparably less tau pathology in the external part of the globus pallidus, lateral aspect of the putamen, and thalamus. The mildly atrophic substantia nigra, locus coeruleus, and pontine base contained widespread tau pathology in the form of neuropil threads and tangles. In the cerebellum, there were moderate numbers of pretangles and neuropil threads in the dentate nucleus, with no apparent tau pathology in the cerebellar cortex or white matter. Histological examination also showed Aβ parenchymal pathology corresponding to Thal phase 2 and CERAD score 0. Assessment of the neurofibrillary tangle tau pathology and corresponding Braak and Braak stage was not possible because of the severity of primary tauopathy. There was no evidence of additional α-synuclein or TDP-43 pathology.

## 4 Discussion

We have described 2 novel *MAPT* mutations presenting as FTD with both behavioral symptoms and prominent semantic impairment. Although segregation data are not available for either mutation, both mutations are predicted to be pathogenic, and importantly both are associated with a primary tauopathy at postmortem.

These patients presented with FTD with behavioral symptoms and cognitive deficits including semantic impairment; previous reports of the phenotype of *MAPT* mutations are consistent with this, for example, in a large series from Manchester, many patients had associated semantic impairment (evidenced by anomia, semantic naming errors, and impaired word and/or object comprehension) in conjunction with behavioral change (Snowden et al., 2015). Both of the patients had features of episodic memory impairment; although unusual for FTD, amnestic syndromes are seen in carriers of *MAPT* mutations, for example, R406W (Tolboom et al., 2010), Q351R (Liang et al., 2014), and duplications (Rovelet-Lecrux et al., 2010). The magnitude of episodic memory impairment can mirror that of Alzheimer's disease (AD), and therefore it is not uncommon for clinical AD to be diagnosed initially in some patients with *MAPT* mutations. Many patients will develop parkinsonian features, including features consistent with a CBS phenotype (or less commonly a PSP phenotype) (Benussi et al., 2015), although this was not seen in our 2 patients.

Age at onset was 45 years in case 1, with a disease duration of 9 years, whereas age at onset was 47 years in case 2, with a disease duration of 7 years, both of which are consistent with previous reports. In general, patients with an underlying pathogenic *MAPT* mutation have a mean age at onset in the mid-50s (Snowden et al., 2015), although onset can be anywhere between the third to the ninth decade. Some mutations tend to cause a more rapidly progressing disease (e.g., P301L) and others a more slowly progressive phenotype (e.g., R406W), although with many cases, such as the ones described here, disease duration lies somewhere in between these 2 extremes.

Although the 2 mutations described here are novel, mutations close to these sites have previously been shown to be pathogenic. Close to the D252V mutation in exon 9 (in case 1), the K257T mutation has been reported to present in the fifth decade with an amnestic and then behavioral syndrome (Rizzini et al., 2000), in keeping with our patient. Similar to the G389_I392del mutation (in case 2), G389R mutations are associated with disease onset between 17–53 years and typically present with a behavioral syndrome with or without parkinsonism (Bermingham et al., 2008, Chaunu et al., 2013, Murrell et al., 1999, Pickering-Brown et al., 2000, Rossi et al., 2008, Tacik et al., 2017).

Typical patterns of atrophy have been identified in large cohort studies of genetic FTD, which suggest an association with relatively symmetrical anterior and medial temporal lobe involvement (Cash et al., 2018, Fumagalli et al., 2018), although asymmetrical bitemporal involvement has also been described (Rohrer et al., 2010), similar to the first case. However, some cases have more frontal involvement such as in our second case (Miki et al., 2018).

Pathologically, both reported cases are 3R-predominant tauopathies with frequent Pick bodies, although in the case 1, there is an additional widespread cortical astrocytic tau pathology with a proportion of astrocytes showing immunoreactivity with 4R-tau. As mentioned before, the closest mutation to D252V is the K257T mutation which has been shown to have predominantly 3R-tau similar to Pick's disease (Forrest et al., 2018) as has the G389R mutation (Pickering-Brown et al., 2000).

Although we do not have segregation data from the families, 2 main factors argue in favor of the pathogenicity of these variants: both are novel (not present in gnomAD, other databases, or in our in-house exome sequencing data on >3000 samples including healthy controls and non-FTD diagnoses); and both have CADD scores of 21.5 indicating these are predicted to be in the top 1% of the most deleterious variations in the human genome.

In conclusion, these 2 novel variants are likely to be pathogenic, but the genetic analysis of other family members to establish segregation of the variants with the disease in the families would be essential to establish pathogenicity and the biological analysis of the function of the mutated protein could also provide important information regarding the role of these variants in the disease process.

## Disclosure

The authors do not have any conflicts of interest.

## Appendix A Supplementary data

Data Profile
